# Low-Proliferative Invasive Mucinous Carcinoma of the Breast in an Octogenarian: A Case Report Highlighting Pathology-Guided De-escalation

**DOI:** 10.7759/cureus.105819

**Published:** 2026-03-25

**Authors:** Rae-Anne Kastle, Christopher M Ahmad, Rachana Tadakamalla, Hima Patel, Amer Abboud

**Affiliations:** 1 General Surgery, Kansas City University, Joplin, USA; 2 Internal Medicine, Kansas City University, Joplin, USA; 3 Pathology, Kansas City University, Joplin, USA; 4 Pathology and Laboratory Medicine, Humboldt Park Health, Chicago, USA

**Keywords:** breast cancer in the elderly, de-escalated treatment pathways, er-positive breast cancer, invasive mucinous carcinoma, ki-67 index

## Abstract

Invasive mucinous carcinoma (IMC) is an uncommon histologic subtype of breast cancer characterized by abundant extracellular mucin and generally indolent behavior. It occurs more frequently in older women and is often associated with favorable prognostic features.An 84-year-old woman with no prior history of breast malignancy was found to have a suspicious lesion in the upper outer quadrant of the left breast on routine imaging. Ultrasound-guided core needle biopsy demonstrated IMC, Grade I/III, involving all four cores, with the largest contiguous tumor focus measuring 1.3 cm. Immunohistochemistry showed strong estrogen receptor positivity (95%), progesterone receptor positivity, human epidermal growth factor receptor 2 negativity (1+), and an extremely low Ki-67 proliferation index (~1%). Pathologic processing met established fixation standards.This case highlights the importance of histologic subtype and proliferative activity in guiding risk stratification and management considerations for breast cancer in elderly patients. Recognition of indolent tumor biology may help avoid overtreatment while maintaining oncologic safety.

## Introduction

Mucinous carcinoma of the breast is a rare variant of invasive ductal carcinoma (IDC), accounting for approximately 1-4% of invasive breast malignancies [1]. Histologically, it is defined by clusters of malignant epithelial cells suspended within abundant extracellular mucin. Mucinous carcinoma is further subclassified into pure and mixed subtypes, which carry distinct prognostic implications. Pure mucinous carcinoma is defined by tumors composed of greater than 90% extracellular mucin, whereas mixed mucinous carcinoma contains an admixed component of conventional IDC [2].

This distinction is essential for accurate risk stratification, as pure mucinous carcinomas are associated with lower rates of lymph node metastasis, reduced recurrence risk, and improved overall survival compared to mixed variants [1]. Reported rates of axillary nodal involvement in pure mucinous carcinoma are approximately 10-15%, whereas mixed subtypes demonstrate substantially higher rates of nodal metastasis [3]. Accordingly, accurate histopathologic classification plays a central role in prognosis and treatment planning, particularly in elderly patients, where therapeutic de-escalation may be considered.

Clinically, invasive mucinous carcinoma (IMC) tends to present in older, postmenopausal women and is frequently associated with hormone receptor positivity, low proliferative indices, and favorable outcomes compared to conventional IDC [4]. However, accurate histologic diagnosis remains essential, as management decisions, particularly in elderly patients, are increasingly informed by tumor biology rather than size alone [4]. These tumors typically exhibit a luminal A molecular subtype, which is highly responsive to endocrine therapy rather than cytotoxic chemotherapy, underscoring the importance of tumor histopathology in treatment decisions [5]. Despite its generally indolent behavior, mucinous carcinoma poses unique diagnostic challenges. The high mucin content influences mammographic density and appearance, potentially leading to benign lesions being misinterpreted and the malignant potential being underestimated. Features such as smooth or irregular margins, signal intensity, and enhancement patterns are also useful in imaging diagnostics [6]. However, tissue diagnosis with core needle biopsy remains critical for definitive diagnosis.

In elderly patients, management decisions require careful integration of factors such as tumor histology, life expectancy, and comorbidities to assess oncologic safety and risks. Guidelines emphasize an individualized approach to treatment in this population. Management decisions must take into consideration geriatric assessments, patient preferences, and competing mortality risks [7]. This report presents a case of low-grade IMC diagnosed on core needle biopsy in an octogenarian, emphasizing the role of pathology in treatment planning.

## Case presentation

An 84-year-old woman with no prior history of breast cancer presented for an evaluation after routine breast imaging identified a suspicious lesion in the upper outer quadrant of the left breast. The patient was managed at Humboldt Park Health in Chicago, Illinois, USA. She denied breast pain, skin changes, nipple discharge, or awareness of a palpable mass. Clinical breast examination was unremarkable.

**Figure 1:**
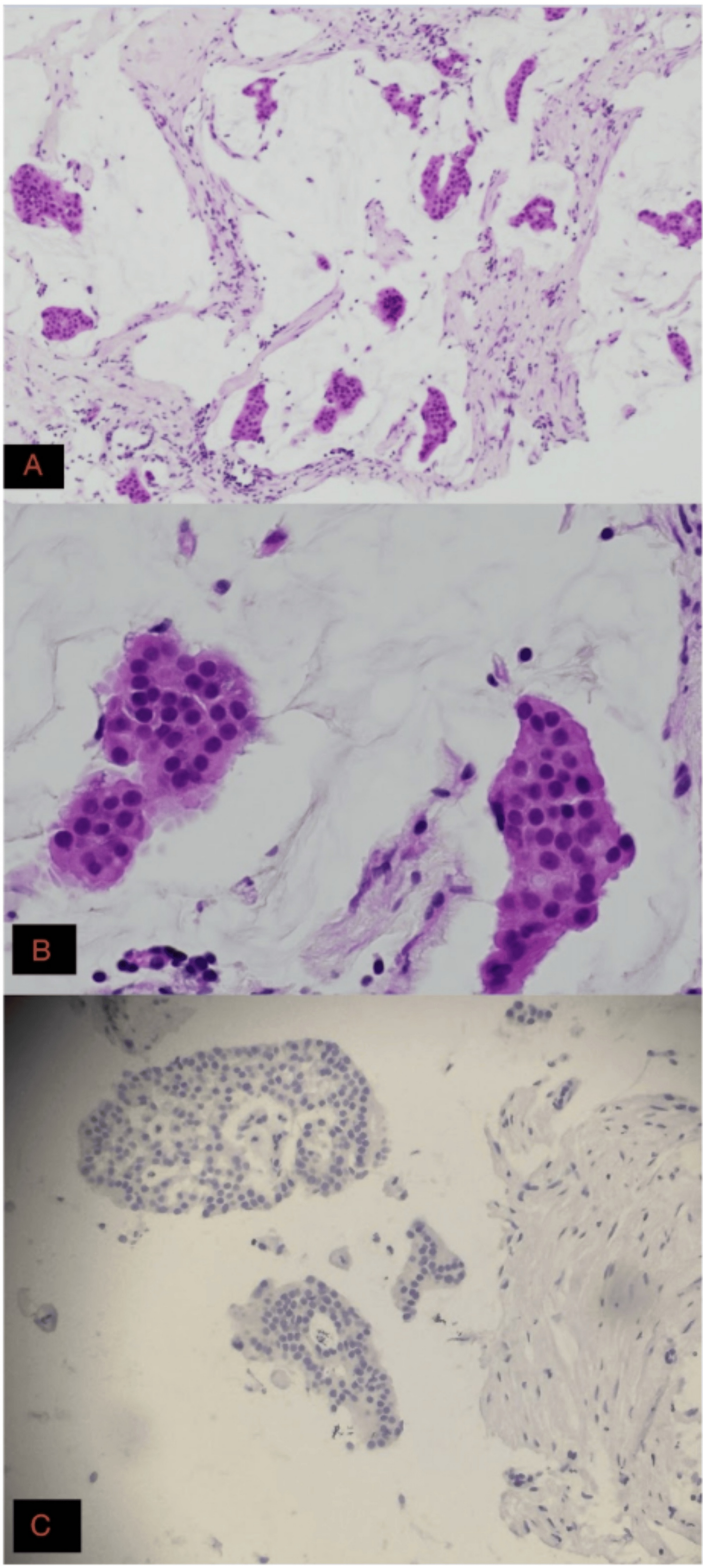
Hematoxylin and eosin–stained sections of invasive mucinous (colloid) carcinoma of the breast. (A) Tumor cell clusters floating within abundant extracellular mucin pools, demonstrating the characteristic architecture of mucinous carcinoma (100× total magnification). (B) Higher-power view showing cohesive nests of malignant epithelial cells with relatively uniform nuclei suspended in mucin (400× total magnification). (C) Lower-power view illustrating multiple mucin pools containing well-demarcated tumor islands (40× total magnification).

Ultrasound-guided core needle biopsy was performed, yielding four cores of tissue. Gross examination revealed pale pink-tan tissue with focal hemorrhagic fragments measuring 7 × 0.3 × 0.2 cm, with the longest individual core measuring approximately 0.5 × 0.3 × 0.2 cm. Gross specimen photography was not available at the time of processing. Histologic evaluation with hematoxylin and eosin (H&E) staining demonstrated classic mucinous architecture (Figure 1). The tumor was characterized by abundant extracellular mucin pools, containing small clusters of malignant epithelial cells with low nuclear grade (Figure 1). The largest contiguous focus of invasive carcinoma measured 1.3 cm. No lymphovascular invasion was identified in the sampled tissue.

**Figure 2:**
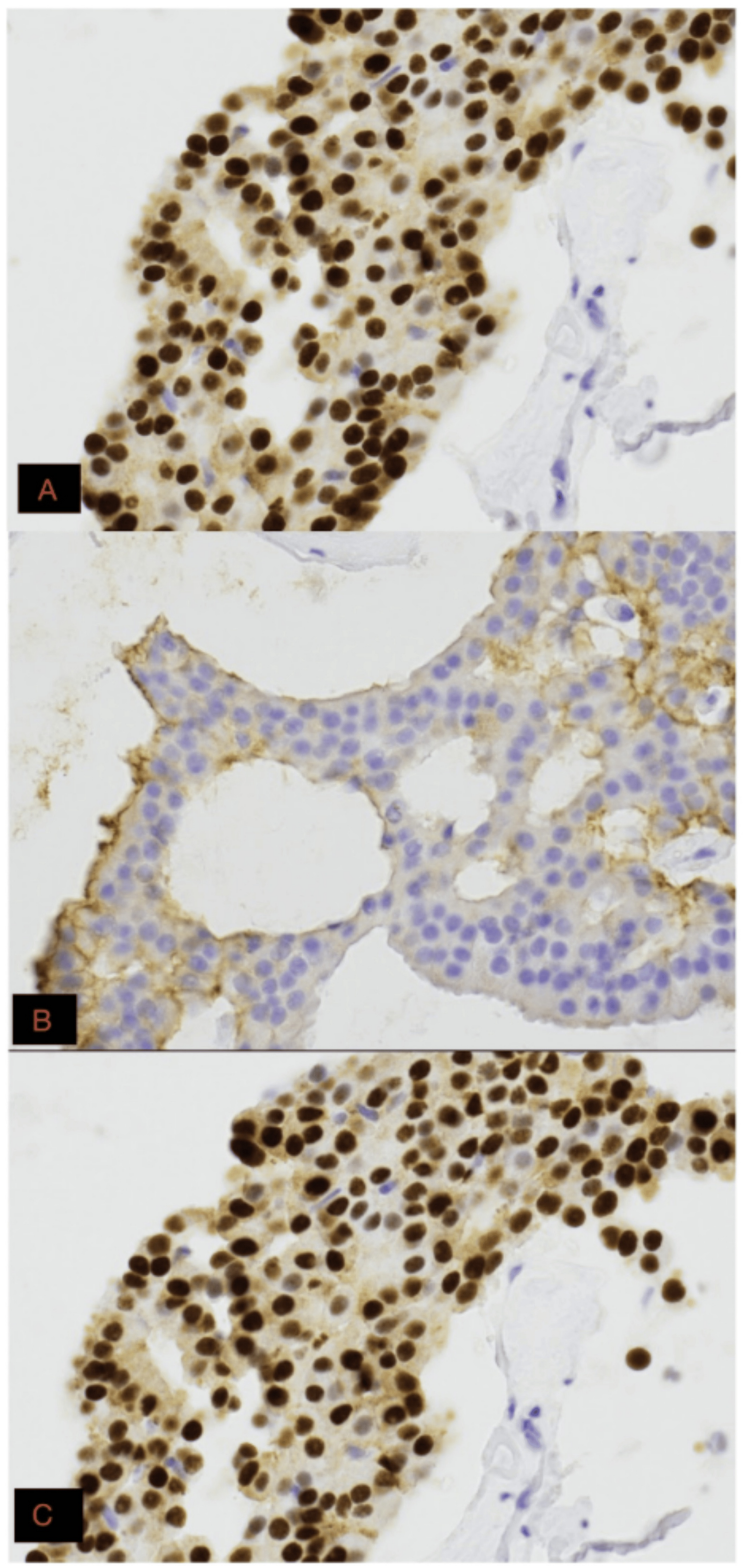
Immunohistochemical characterization of mucinous breast carcinoma. (A) Estrogen receptor (ER) immunohistochemistry demonstrating strong diffuse nuclear staining in tumor cells (400× total magnification). (B) Human epidermal growth factor receptor 2 (HER2) immunohistochemistry showing absent to faint incomplete membranous staining (400× total magnification). (C) Progesterone receptor (PR) immunohistochemistry demonstrating strong diffuse nuclear staining consistent with PR positivity (400× total magnification).

**Figure 3:**
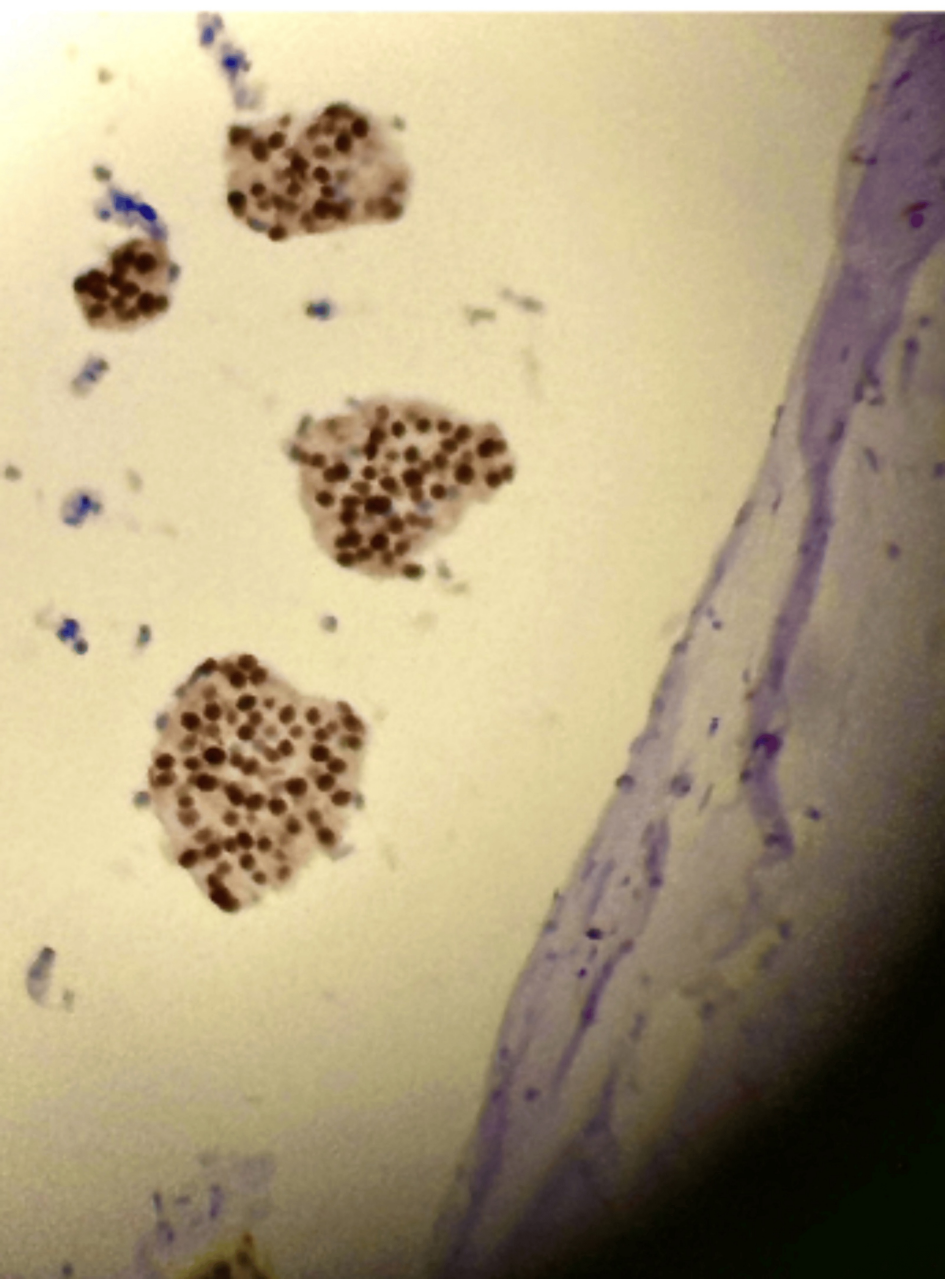
Ki-67 proliferation index. Ki-67 immunohistochemical staining demonstrates low proliferative activity with approximately 1% nuclear positivity (400× total magnification).

Immunohistochemical (IHC) analysis revealed strong estrogen receptor (ER) expression in approximately 95% of tumor cells, progesterone receptor (PR) positivity, human epidermal growth factor receptor 2 (HER2) negativity (score 1+), and a Ki-67 proliferation index of approximately 1% (Figures 2, 3; Table 1). Immunohistochemistry demonstrated diffuse nuclear staining consistent with PR positivity. Specimen fixation in 10% neutral buffered formalin was initiated promptly following tissue acquisition, with a documented cold ischemia time of approximately five minutes. Fixation duration exceeded six hours but was less than 72 hours, meeting recommended processing standards.

**Table 1:** Pathologic and immunohistochemical features driving management decisions. cm: centimeter; HER2: human epidermal growth factor receptor 2; ER: estrogen receptor; PR: progesterone receptor; ASCO: American Society of Clinical Oncology; CAP: College of American Pathologists

Feature	Findings
Patient age	84 years
Tumor location	Left breast, upper outer quadrant
Specimen type	Ultrasound-guided core needle biopsy
Number of cores involved	4 of 4 cores
Histologic type	Invasive mucinous carcinoma
Histologic grade (Nottingham)	Grade I / III
Predominant architecture	Clusters of malignant epithelial cells suspended in abundant extracellular mucin
Largest contiguous invasive focus	1.3 cm
Lymphovascular invasion	Not identified
ER	Positive (≈95% nuclear staining)
PR	Positive (diffuse nuclear staining)
HER2	Negative (1+ by immunohistochemistry)
Ki-67 proliferation index	Approximately 1%
Molecular surrogate subtype	Luminal A–like
Cold ischemia time	5 minutes
Fixation type	10% neutral buffered formalin
Fixation duration	>6 hours and <72 hours
Specimen processing	Met ASCO/CAP guideline recommendations

Final pathology confirmed IMC with low-grade features and extremely low proliferative activity. Additional treatment decisions and follow-up outcomes were not available at the time of reporting. Key histopathological features identified on biopsy are summarized in Table 2.

**Table 2:** Histopathological features identified on core needle biopsy. This table details the histomorphological characteristics of the biopsied tissue. The presence of invasive mucinous carcinoma is characterized by significant extracellular mucin production, which often correlates with specific radiographic appearances on CT and MRI. The low-to-intermediate nuclear grade and the absence of identified lymphovascular invasion are important prognostic indicators. ER: estrogen receptor; PR: progesterone receptor; HER2: human epidermal growth factor receptor 2

Feature	Details
Histologic type	Invasive mucinous carcinoma
Key microscopic feature	Abundant extracellular mucin with malignant epithelial clusters
Nuclear grade	Low-to-intermediate
Lymphovascular invasion	Not identified
Receptor status	ER-positive, PR-positive, HER2-negative

## Discussion

This case illustrates how detailed histopathologic characterization, particularly mucinous subtyping and proliferative index, can directly inform management considerations in very elderly patients with breast cancer. IMC of the breast is a rare histologic variant, representing approximately 1% to 4% of all invasive breast malignancies [8]. It is most frequently diagnosed in postmenopausal women, with a peak incidence in the seventh and eighth decades of life. This case of an 84-year-old female patient presents the "classic" demographic and pathologic profile of the disease. Yet, it is characterized by an exceptionally low proliferative index, warranting a review of current management strategies in older adults.

Histopathologic subtyping and prognosis

The primary diagnostic challenge and prognostic indicator in IMC is the distinction between pure and mixed subtypes. According to established criteria, a diagnosis of "pure" mucinous carcinoma requires that the mucinous component account for at least 90% of the tumor [3]. In our patient, the core biopsy demonstrated exclusive mucinous architecture across all four sampled cores (Figure 1). While definitive classification as pure mucinous carcinoma requires excisional pathology, the uniform mucinous architecture across all sampled cores strongly supports a pure subtype.

The clinical significance of this distinction cannot be overstated; pure mucinous carcinomas are associated with significantly lower rates of axillary lymph node involvement (often <10%) and a specialized indolent clinical course compared to mixed mucinous carcinoma or conventional IDC [9]. Ten-year survival rates for the pure subtype frequently exceed 90%, making it one of the most favorable histologic variants of breast cancer [3]. Lymphovascular invasion was not identified in this case.

In contrast, conventional IDC is more often associated with higher proliferative activity, as well as an increased likelihood of lymphovascular invasion and nodal metastasis, contributing to a more aggressive clinical course. These features frequently necessitate multimodal treatment strategies, including chemotherapy and comprehensive surgical staging. The relatively favorable biology of mucinous carcinoma, particularly in its pure form, underscores the importance of distinguishing these entities, as management approaches used for conventional IDC may not be directly applicable.

The biological profile: ER, PR, and Ki-67

The IHC profile of this case, strongly ER-positive, HER2-negative, and low-grade, aligns with a luminal A molecular subtype. The tumor demonstrated PR positivity, further supporting a luminal A-like molecular profile. Notably, even in cases where PR expression is absent, the prognosis of pure mucinous carcinoma remains favorable when strong ER expression is preserved [10].

At the molecular level, mucinous carcinomas are typically associated with a luminal A-like profile, characterized by strong hormone receptor expression, HER2 negativity, and low proliferative activity. This biologic pattern is consistent with endocrine-responsive disease and an overall indolent clinical course compared to more aggressive breast cancer subtypes. These features further support a treatment approach that prioritizes endocrine therapy over cytotoxic interventions in appropriately selected patients.

The most notable finding in this case is the Ki-67 of ~1%. Ki-67 is a nuclear marker of cellular proliferation; while IMC typically exhibits lower Ki-67 levels than conventional IDC, a 1% index represents the extreme end of the indolent spectrum. Ki-67, while widely used as a marker of cellular proliferation, is subject to significant interobserver variability and lacks universally standardized cutoffs across clinical practice. Reported thresholds distinguishing low versus high proliferative activity vary across studies and institutional protocols, particularly within luminal breast cancer subtypes. In mucinous carcinoma specifically, Ki-67 indices are generally lower than those observed in conventional IDC, often falling within a low to intermediate range. However, a proliferation index approximating 1% represents an extreme on the indolent spectrum and is infrequently reported. In this context, the observed Ki-67 value further supports a biologically low-risk tumor profile, though it should be interpreted alongside histologic subtype and receptor status rather than in isolation [11]. This exceptionally low proliferative activity suggests a tumor with negligible mitotic turnover, which directly informs the risk-benefit analysis of aggressive surgical and adjuvant interventions [12].

Management in the very elderly

The management of an octogenarian with a 1.3 cm, low-proliferative, pure mucinous tumor involves balancing oncologic safety with the risks of overtreatment. In the context of "Choosing Wisely" initiatives and geriatric oncology, this case supports consideration of treatment de-escalation.

In this case, the combination of pure mucinous architecture, absence of lymphovascular invasion within sampled tissue, strong hormone receptor positivity, HER2 negativity, and an exceptionally low Ki-67 index collectively suggests a tumor with minimal proliferative and metastatic potential, consistent with the reported favorable biology and low rates of nodal involvement in pure mucinous carcinoma [13]. These features provide a biologic rationale for considering less aggressive management strategies, including the potential omission of certain surgical or adjuvant interventions in carefully selected patients. However, given that these conclusions are derived from core needle biopsy without complete pathologic staging, such considerations must be approached cautiously and integrated with comprehensive clinical and radiologic assessment.

Regarding axillary surgery, patients over 70 years of age with clinically node-negative, ER-positive, HER2-negative tumors, omitting sentinel lymph node biopsy is increasingly considered an acceptable standard, as it rarely alters the course of adjuvant systemic therapy [14]. Adjuvant radiation in landmark trials, such as CALGB 9343, has demonstrated that in women aged 70 and older with early-stage, ER-positive breast cancer, the omission of radiotherapy after breast-conserving surgery does not significantly impact overall survival or the risk of distant metastasis, despite a modest increase in local recurrence [15]. This case illustrates that detailed pathology, specifically the Ki-67 index and mucinous subtyping, is essential for identifying patients who can safely bypass the morbidity of surgery or radiation.

This case reinforces the idea that, in very elderly patients, the tumor's biological behavior, rather than its size alone, should dictate the aggressiveness of care. The identification of a pure mucinous subtype with a near-minimal Ki-67 index provides the clinical confidence required to pursue de-escalated treatment pathways, ensuring that the patient's quality of life is preserved without compromising oncologic outcomes. These findings should be interpreted within the limitations of biopsy-based diagnosis, as definitive treatment planning requires correlation with surgical pathology, nodal evaluation, and full staging workup.

Limitations

This report is limited by its single-patient design and reliance on core needle biopsy rather than definitive surgical excision. Although all sampled cores demonstrated uniform mucinous architecture suggestive of a pure subtype, complete tumor evaluation would be required to definitively exclude a mixed component. Axillary nodal status was not surgically assessed, and lymphovascular invasion was evaluated only within the sampled tissue, resulting in incomplete pathologic staging. Additionally, treatment decisions and longitudinal follow-up data were not available at the time of reporting, preventing a direct assessment of recurrence risk or long-term outcomes associated with potential de-escalation of management. Finally, while pre-analytic processing met established standards, Ki-67 assessment is subject to known interobserver variability. As with all case reports, generalizability is limited, and conclusions should be interpreted within the context of individualized clinical decision-making rather than as practice-changing evidence.

## Conclusions

IMC of the breast represents a distinct histologic subtype characterized by abundant extracellular mucin and generally favorable biologic behavior. Recognition of this subtype, along with careful evaluation of hormone receptor status and proliferative activity, contributes meaningfully to risk stratification, particularly in elderly patients. However, in this case, conclusions regarding treatment de-escalation must be interpreted with caution. The diagnosis and biologic assessment were based solely on core needle biopsy without definitive surgical excision, limiting complete evaluation of tumor heterogeneity, margin status, and the presence of a potential mixed component. Additionally, axillary nodal status was not assessed, and comprehensive staging data, including imaging correlation, were not available. These factors restrict the ability to fully characterize disease extent and oncologic risk. Accordingly, while the observed low-grade morphology and extremely low proliferative index suggest an indolent tumor biology, management decisions should not rely solely on biopsy findings. This case instead highlights the importance of integrating histopathologic features with complete clinical, radiologic, and surgical evaluation when determining appropriate treatment strategies in elderly patients.
